# Biochemical and Bioinformatic Studies of Mutations of Residues at the Monomer–Monomer Interface of Human Ornithine Aminotransferase Leading to Gyrate Atrophy of Choroid and Retina

**DOI:** 10.3390/ijms24043369

**Published:** 2023-02-08

**Authors:** Fulvio Floriani, Carla Borri Voltattorni, Barbara Cellini, Riccardo Montioli

**Affiliations:** 1 Department of Neurosciences, Biomedicine and Movement Sciences, Section of Biological Chemistry, University of Verona, 37134 Verona, Italy; 2 Department of Medicine and Surgery, University of Perugia, 06123 Perugia, Italy

**Keywords:** ornithine aminotransferase, pyridoxal 5′-phosphate, pathogenic variants, gyrate atrophy of choroid and retina, interface mutations

## Abstract

Deficit of human ornithine aminotransferase (hOAT), a mitochondrial tetrameric pyridoxal-5′-phosphate (PLP) enzyme, leads to gyrate atrophy of the choroid and retina (GA). Although 70 pathogenic mutations have been identified, only few enzymatic phenotypes are known. Here, we report biochemical and bioinformatic analyses of the G51D, G121D, R154L, Y158S, T181M, and P199Q pathogenic variants involving residues located at the monomer–monomer interface. All mutations cause a shift toward a dimeric structure, and changes in tertiary structure, thermal stability, and PLP microenvironment. The impact on these features is less pronounced for the mutations of Gly51 and Gly121 mapping to the N-terminal segment of the enzyme than those of Arg154, Tyr158, Thr181, and Pro199 belonging to the large domain. These data, together with the predicted ΔΔG values of monomer–monomer binding for the variants, suggest that the proper monomer–monomer interactions seem to be correlated with the thermal stability, the PLP binding site and the tetrameric structure of hOAT. The different impact of these mutations on the catalytic activity was also reported and discussed on the basis of the computational information. Together, these results allow the identification of the molecular defects of these variants, thus extending the knowledge of enzymatic phenotypes of GA patients.

## 1. Introduction

Human ornithine δ-aminotransferase (hOAT) is a pyridoxal 5-phosphate (PLP)-dependent enzyme that catalyzes the transfer of the δ-amino group of L-ornithine (L-Orn) to α-ketoglutarate (αKG) yielding glutamate δ-semialdehyde (GSA) and L-glutamate in the mitochondrial matrix. The product GSA spontaneously cyclizes forming Δ1-pyrroline-5-carboxylate (P5C), an important precursor of the proline synthesis. hOAT is a nuclear encoded mitochondrial protein synthetized as a 49 kDa precursor, which is then processed into the mature enzyme of ≈45 kDa upon mitochondrial internalization [1,2]. On the basis of the structural features, hOAT belongs to the aminotransferases subgroup II of the Fold-type I group of PLP-dependent enzymes together with γ-aminobutyric acid aminotransferase and ω-amino acid pyruvate aminotransferase [3]. The crystal structure revealed that hOAT monomers are composed by 12 α-helices and 14 β-sheets organized in three domains: a N-terminal segment (residues 25–95), a large domain (residues 96–345) and a C-terminal small domain (residues 346–439) [4]. Two monomers are associated in a tight homodimer through a wide surface of interactions involving the large and the N-terminal domains of both subunits. As for all Fold-type I enzymes, the hOAT active sites are generated by the dimerization process and are composed by residues belonging to both the monomers. Although chromatographic and ultracentrifugation analyses on the recombinant hOAT revealed that, unlike the majority of the PLP-dependent enzymes, the dimeric units of the enzyme are assembled in a homotetrameric structure [5], it was found that the dimer is fully competent. In fact, when Arg217, a key interchain element supporting important contacts between the dimeric units, was mutated into alanine, the tetramerization process was impaired but the enzyme retained full catalytic efficiency. Moreover, the tetramer–dimer equilibrium of hOAT depends on the cofactor binding, as indicated by the equilibrium of dissociation constant (K_Dtet-dim_) of holoOAT that is five-fold lower than the one of apoOAT. This strongly suggests that the PLP binding is able to shift the K_Dtet-dim_ value [5].

The deficit of hOAT is known to be the cause of the gyrate atrophy of choroid and retina (GA), a rare autosomal recessive disorder mainly characterized by a progressive degeneration of the choroid and retina tissues leading to the loss of vision in a few decades [6]. To date, the HGMD database (http://www.hgmd.cf.ac.uk, accessed on 12 December 2022) lists 70 pathogenic mutations of the *OAT* gene causing GA, 58% of which are missense mutations [7]. In the past years, molecular studies on some recombinant purified pathogenic variants of hOAT revealed a wide spectrum of possible effects of the missense pathogenic mutations on the structural and functional properties of the enzyme including catalysis, coenzyme binding, oligomerization and folding [8,9,10]. Among the characterized pathogenic variants, the R154L showed peculiar features [10]. In fact, although the Arg154 is located at the monomer–monomer interface far from the active site and is not directly involved in the dimer–dimer interface, the R154L enzyme was found to be almost completely inactive with alterations of the chiral microenvironment of the cofactor and unable to generate the tetrameric structure at least up to 10 μM protein concentration [10]. On the basis of these findings, in this work we decided to gain insight into the effect(s) of the known monomer–monomer interfacial pathogenic mutations of hOAT present in homozygous or hemizygous patients. The reason is that the expression system allows to purify and characterize the homotetrameric and/or homodimeric variants in their recombinant form, thus providing reliable information on the enzymatic phenotypes of patients bearing a single missense mutation. About 10 of the known missense pathogenic mutations of OAT concern residues mapping to the monomer–monomer interface [7]. Six of them were identified in homozygosis or hemizygosis patients. Thus, in addition to the R154L [11], here, we take into consideration five more pathogenic variants involving the interfacial residues (G51D [12], G121D [12], Y158S [13], T181M [14] and P199Q [15]) by a combination of spectroscopic, kinetic, chromatographic and bioinformatic analyses.

The results obtained revealed that the R154L, Y158S, T181M, and P199Q mutations, concerning residues located in the large domain, cause a change in the thermal stability, the K_Dtet-dim_ value and the PLP microenvironment that is more pronounced than that showed in the G51D and G121D mutations involving residues lying in the N-terminal segment. A correlation between the tetramer↔dimer equilibrium of these variants and their predicted ΔΔG value of monomer–monomer binding was proposed and discussed. Moreover, while the Y158S, T181M, and P199Q variants show a transaminase activity lower (ranging from 5- to 130-fold) than that of the wild-type enzyme, the R154L variant does not exhibit detectable activity. On the other hand, the G51D and G121D substitutions cause a rapid loss of catalytic activity making quite difficult kinetic studies from initial velocity. Overall, these studies highlight the different relevance of the interfacial monomer–monomer residues on the structural and/or functional features of hOAT and point out the pathogenesis of their mutations associated with GA.

## 2. Results and Discussion

### 2.1. In Silico Analyses of hOAT Interfaces and of the Mutation Sites

**Figure 1:**
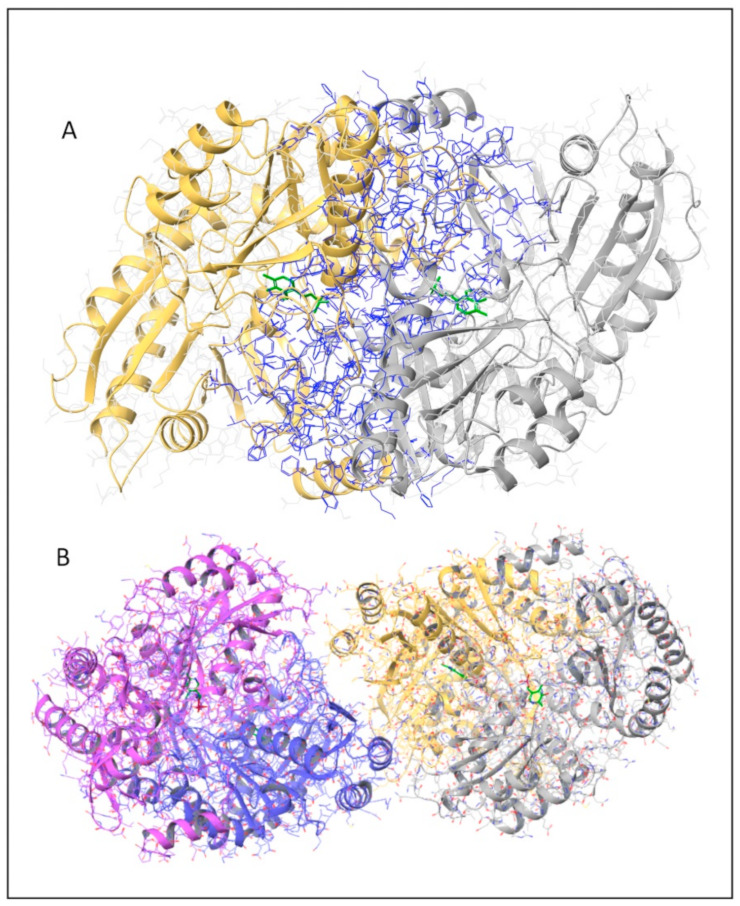
Dimeric and tetrameric hOAT structures. Cartoon representation of human hOAT structure; the protein chains are differently colored and the PLP molecules are represented as green sticks. (**A**) OAT dimeric structure in which all the interface residues are highlighted in blue. (**B**) OAT tetrameric structure in which the two dimers are colored magenta/blue and yellow/gray, respectively. The image was generated by Maestro v13.3 software (Schrödinger, New York, NY, USA).

Both the monomer–monomer interface of the dimeric hOAT unit and the dimer–dimer interface of the tetrameric assembly of hOAT were analyzed by means of the PDBePISA online tool and Maestro software v13.3 (Schrödinger). The monomer–monomer surface of contacts is particularly extended and resulted 5200 Å^2^, ~28% of the total monomer surface (Figure 1A). Such large area includes both polar and hydrophobic residues contributing to the inter-chain contacts. On the other hand, the dimer–dimer interface surface area resulted to be only 860 Å^2^, ~3.5% of the total monomer surface, and includes mainly polar residues. It is noteworthy that in the hOAT tetramer, each of the junction regions between hOAT dimers involve only one subunit of a dimer and both the monomers of the neighboring dimer (Figure 1B). On the basis of these observations, it seems possible to suggest that the correct match between the monomers in the dimeric unit formation could be important not only for the dimerization but also for the tetramerization of hOAT.

**Figure 2:**
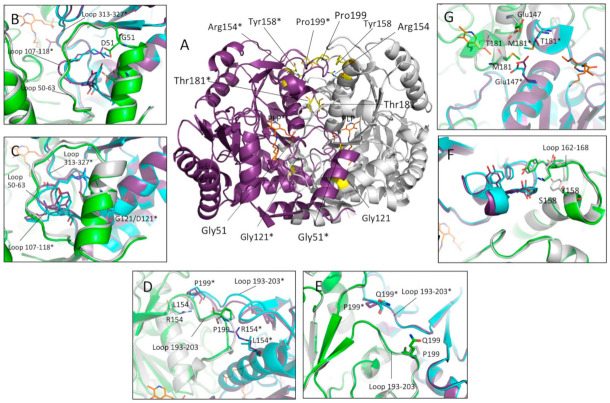
In silico analysis of the position and possible local effects of the mutations. The dimeric structure of hOAT is represented in cartoon style and the PLP-Lys292 complex is represented as orange sticks. Monomers are differently colored as grey/purple and green/cyan for the wild-type and mutated hOAT structures, respectively. Panel (**A**), representation of the structural position of the residues subjected to mutation on the hOAT wild-type structure, the mutation sites are indicated and highlighted as yellow sticks. Panels (**B**–**G**), over imposition of the OAT wild-type structure and the mutated structures of (**B**) G51D, (**C**) G121D, (**D**) R154L, (**E**) P199Q, (**F**) Y158S and (**G**) T181M, obtained by in silico mutagenesis. * Residues belonging to the neighboring subunit. Image was obtained by PyMol software (Schrödinger, New York, NY, USA).

By mapping the mutation sites of the homozygous and hemizygous missense mutations of hOAT on the enzyme crystal structure, we found that six of them, G51D, G121D, R154L, Y158S, T181M and P199Q, involve residues located at the monomer–monomer interface (Figure 2A). At first, the software BindProfX was used to calculate the predicted ΔΔG of interaction of the monomers in the dimeric unit caused by each interface mutation (Table 1). Results showed that, even if to a different extent, all the selected mutations are predicted to affect the free energy of binding of the monomers. In addition, the three residues, Arg154, Thr181 and Pro199, were highly conserved, while Gly51, Gly121 and Tyr158 have a medium-low conservation level, as evaluated by the ConSurf server.

The six mutations involve residues located in different protein regions (Figure 2A). In particular, Gly51 and Gly121 are located at the interface of the N-terminal region of one monomer and the large domain of the neighboring subunit. Gly51 is located between the N-terminal α-helix 40–49 and the loop 50–63, a flexible region at the entrance of the active site, while Gly121 lies on the 119–131 α-helix of the neighboring subunits. At the local level, both the G51D and G121D substitutions are predicted to affect the conformation of the interface loop 107–118 and the relative position of the 40–49 and 119–131 helices, possibly affecting the active site loop 313–327 (Figure 2B,C). Arg154 and Pro199 belong to the large domain and are directly contacting each other; indeed, the Nε of Arg154 of one subunit engages, by a hydrophobic bond, the backbone oxygen of Pro199 of the neighboring subunit (Figure 2D). The drastic substitution Arg154→Leu introduces a non-polar residue into a polar cleft and abolishes an inter-chain contact between monomers. On the whole, the perturbation is predicted to affect the conformation of the interface loop 193–203 (Figure 2D). On the other hand, the replacement of Pro199 with the bulkier Gln residue is expected to generate steric hindrance at the monomer–monomer interface; however, the in silico mutagenesis suggests that the Gln199 side chains should turn toward the surface, generating minimum bulk between the monomers. Based on this prediction, it is reasonable to suggest that the loss of a proline residue could induce a backbone relaxation of the interface loop 193–203 (Figure 2E). On the whole, the R154L and P199Q are expected to have a similar structural impact. Tyr158 belongs to the α-helix 142–160 and is placed near to the Pro199-Arg154 interaction. The π-system of Tyr158 contributes to hydrophobic interchain contacts and to the conformation of the surface loop 162–168 which, in turn, is involved in multiple interchain interactions (Figure 2F). The substitution with a Ser residue compromises the structural role of the residue by abolishing all the hydrophobic contacts. However, the ΔΔG of dimerization of the Y158S variant measured by BindProfX server may be underestimated (see below), since the major impact of this mutation on the monomers interface could be indirect and could involve the interface residues belonging to loop 162–168 (Figure 2F). In the hOAT dimer, Thr181 of one subunit faces the same residue of the neighboring subunit in the region between the two active sites (Figure 2A). Thr181 is hydrogen-bonded to the Glu147 side chain and is located close to Arg180, a residue known to be critical for catalysis [9]. The T181M in silico substitution suggests that the bulky Met side chain, in addition to the loss of the contact with Glu147, generates steric hindrance between monomers that in turn might force the relocation of the nearby side chains (Figure 2G). Moreover, the proximity to the Arg180 suggests that the T181M substitution might also affect the catalytic activity.

### 2.2. Expression and Purification of the hOAT Interface Variants

**Figure 3:**
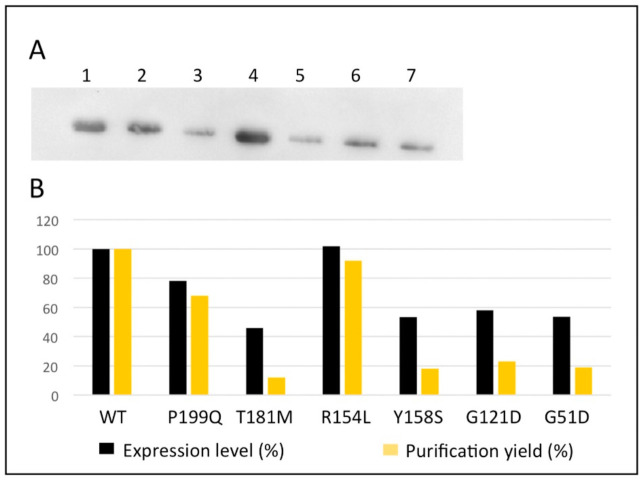
Expression level and yield of purification of the OAT variants. (**A**) Western Blot analysis of the soluble fraction of the *E. coli* cells lysate expressing (1) hOAT wild-type, (2) P199Q, (3) T181M, (4) R154L, (5) Y158S, (6) G121D and (7) G51D. (**B**) percentage of expression level and yield of purification of the hOAT variants with respect to those of wild-type hOAT. Expression levels were quantified by the ImageJ software on the basis of the mean intensity of the bands of two independent experiments. The percentages of yield of purification of the variants were calculated on the basis of the reported wild-type value of 35 mg/L of culture.

To characterize the structural and functional effect(s) of the interface mutations, expression vectors for the G51D, G121D, Y158S, T181M and P199Q variants were obtained by site directed mutagenesis, while that for R154L was previously generated [10]. The expression level of the variants in soluble fraction of *E. coli* cells lysate was compared with the one of the wild-type hOAT (Figure 3). The quantification of the relative expression indicates that R154L and P199Q variants have slight or no effect on the soluble protein level while the remaining variants, G51D, G121D, Y158S and T181M, exhibit a consistent reduction of the soluble protein. These data suggest that, unlike the R154L and P199Q mutations, the G51D, G121D, Y158S and T181M mutations could affect the folding efficiency of the protein. However, the *E. coli* expression system might be unsuitable to evaluate the impact of the mutations on the folding process of a human mitochondrial enzyme. The recombinant hOAT variants were purified, as reported in the Material and Methods section. All the variants exhibited the expected molecular weight (45 kDa) and with adequate purity level (Figure S1). The yield of purification resulted substantially in line with the expression level (Figure 3).

### 2.3. Impact of the G52D, G121D, R154L, Y158S, T181M and P199Q Mutations on the Spectroscopic Features and Thermal Stability of hOAT

**Figure 4:**
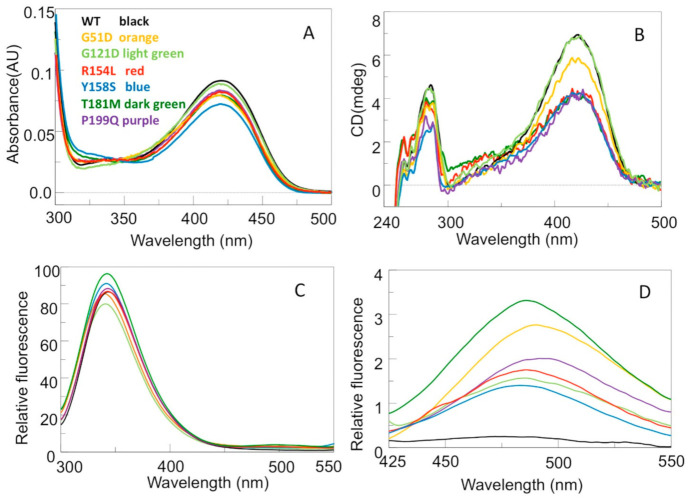
Spectroscopic analysis of the interface pathogenic variants of hOAT. (**A**) Absorption spectra and (**B**) nearUV-vis CD spectra of 6 µM enzyme in Hepes 50 mM pH 8.0, NaCl 150 mM. (**C**) Intrinsic fluorescence emission spectra of 1 µM enzyme upon excitation at 280 nm. (**D**) ANS fluorescence emission spectra of 1 µM enzyme upon excitation at 367 nm. Both intrinsic fluorescence and ANS fluorescence emission spectra were registered in Hepes 50 mM pH 8.0, NaCl 150 mM. All the CD and fluorescence spectra were registered in the presence of 10 µM exogenous PLP. All the panels share the indicated color code.

hOAT displays an absorbance band centered at 420 nm and a shoulder at 340 nm associated with positive dichroic signals at the same wavelengths. Excitation at 420 or 340 nm gives rise to an emission fluorescence spectrum with a maximum around 505–510 nm. On these bases, the 420 and 340 nm bands have been attributed to the ketoenaminic and enoliminic tautomeric forms of the internal aldimine, respectively [5]. In addition, the enzyme displays a positive dichroic band at 284 nm, and, upon excitation at 280 nm, an emission fluorescence maximum at 341 nm [5]. The far-UV CD spectra of the pathogenic variants compared with that of the wild-type hOAT indicate that the examined mutations do not affect the overall secondary structure of the enzyme (Figure S2). To understand if the mutations alter the PLP binding mode, the UV–visible absorbance and CD spectrum of each enzymatic species at 6 μM concentration were registered. Although all the variants exhibit absorbance spectra qualitatively similar to that of the wild-type, the intensity of their 420 nm absorbance bands is, even if to a different extent, lower than that of the wild-type (Figure 4A). On the other hand, as compared to the wild-type, the magnitude of the positive dichroic signal at 420 nm of the G121D variant is unaltered, while that of the remaining variants is reduced, in particular that of the R154L, Y158S, T181M, and P199Q variants (Figure 4B). The optical activity (mdeg/A_420nm_ absorbance unit) was also measured to determine if these variants exhibit similar properties with respect to the asymmetric environment of the bound PLP in the wild-type. The wild-type, G51D, R154L, T181M, P199Q, G121D, and Y158S enzymes have optical activity values of 76.6, 74.3, 48.2, 47.4, 49.0, 77.6, and 60.3 mdeg/A_420_ nm, respectively. Altogether, these data suggest that the chiral environment of the internal aldimine is slightly changed in G51D, unaltered in G121D, while it is strongly changed in the remaining variants.

Furthermore, in order to establish if the pathogenic mutations under study may alter the tertiary structure of hOAT, near-UV CD, intrinsic and ANS fluorescence emission spectra of the variants were acquired. Figure 4B–D show that, with respect to the wild-type enzyme (i) the magnitude of the dichroic signal in the near-UV of all the variants but G121D is lower, (ii) the emission intensity and the maximum of the intrinsic fluorescence of the R154L and Y158S variants are unchanged, while the emission intensity of the T181M and P199Q variants is slightly higher, and the maximum of the intrinsic emission fluorescence of the G51D and G121D variants is about 1 nm blue shifted, and (iii) the ANS emission fluorescence intensity of all the variants studied is higher, ranging from 6- to 30-fold. Taken together, the near-UV CD and the intrinsic emission fluorescence spectra indicate that all the mutations, with the exception of the G121D one, cause structural changes affecting the position and/or orientation of aromatic residues. Such conformational changes are confirmed by the ANS fluorescence, which reveal that all the analyzed mutations cause the exposure of hydrophobic patches on the protein surface. This is probably due to the exposure of hydrophobic interface residues that are masked in the tetrameric structure of the wild-type enzyme.

It is of interest to note that the T181M variant exhibits the major alterations of all the spectroscopic signals and the lower expression level (Figure 3). Since Thr181 is an internal residue located in the center of the dimer and close to the active site (Figure 2A), it is not surprising that that its substitution with a methionine might cause conformational changes of monomer interface residues involving the PLP internal aldimine. On the other hand, the other five mutations involving surface or peripheral interface residues are expected to bring about the structural effects at local level. However, the alteration of the internal aldimine of the G51D, R154L, Y158S, and P199Q variants suggests that the interface perturbation could extend toward the PLP microenvironment.

To verify whether the analyzed mutations could affect the thermal stability of hOAT, we compared the thermal unfolding profiles of the purified variants with that of the wild-type enzyme. Thermal denaturation was studied by monitoring the decrease of the dichroic negative signal at 222 nm, indicative of the loss of the protein secondary structure. Similar to the wild-type enzyme, all the variants are denatured in a single melting process with melting temperatures (T_m_) values reported in Table 1. With respect to the T_m_ value of the wild-type enzyme (67.8 °C), the G51D the G121D variants shows a T_m_ value ~1–3 °C lower, while the R154L, T181M, Y158S, and P199Q variants exhibit the most pronounced alteration with T_m_ values ~10 °C lower. Notably, the comparison of the ΔΔG with the T_m_ values indicates that the variants more sensitive to thermal stress, i.e., R154L, T181M, and P199Q, are associated with high ΔΔG values. This could be true also for Y158S considering that its ΔΔG predicted value could be underestimated (see above). This correlation indicates that more the interaction between monomers is altered, the lower becomes the OAT thermal stability. On the basis of these observations, we may suggest that the initial step of OAT unfolding process should be the monomerization, as previously demonstrated for other PLP-dependent enzymes [16,17]. Interestingly, the R154L, Y158S, T181M, and P199Q variants exhibit the major alterations of the internal aldimine spectra, indicating a possible correlation between the interface perturbation and the PLP microenvironment alteration.

### 2.4. Impact of the Interface Variants on the Quaternary Structure of hOAT

**Figure 5:**
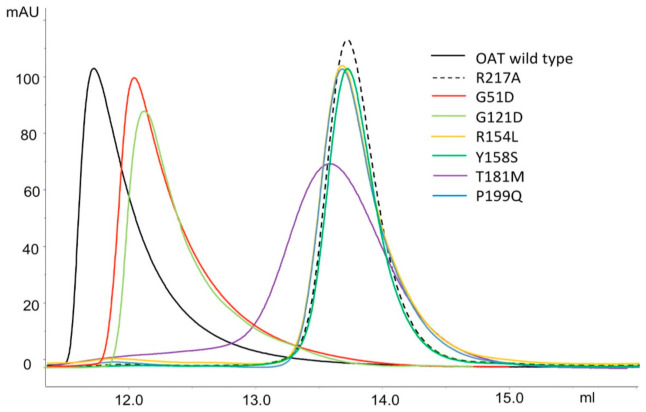
Sec analysis of the quaternary structure. hOAT wild-type, the artificial OAT dimeric variant R217A and the pathogenic variants G51D, G121D, R154L, Y158S, T181M and P199Q were loaded at 10 µM enzyme concentration on a Superdex 200 10/300 Increase column equilibrated in HEPES 50 mM pH 8.0, NaCl 150 mM, 20 µM PLP. Chromatographic profiles were analyzed by Unicorn v7.3 software (GE Healthcare, Chicago, IL, USA).

As previously mentioned, hOAT is present in solution as both homodimer and homotetramer in rapid equilibrium. Such equilibrium is influenced by cofactor binding; indeed, the OAT holoform exhibits a K_Dtet-dim_ value 5-times lower than that of apoOAT [5]. Here, the oligomeric state of the G51D, G121D, R154L, Y158S, T181M and P199Q variants was investigated by analytical SEC up to 10 µM enzyme concentration, in the presence of 20 µM exogenous PLP. As shown in Figure 5, 10 µM wild-type hOAT exhibited an elution volume of 11.7 mL, a value corresponding to the tetrameric form on the basis of the column calibration curve (Section 3). On the other hand, R154L, Y158S, T181M and P199Q at 10 µM enzyme concentration exhibited an elution volume around 13.6 mL, the same value of the OAT artificial dimer R217A [5] and corresponding to the molecular weight of a dimer (Figure 5).

**Figure 6:**
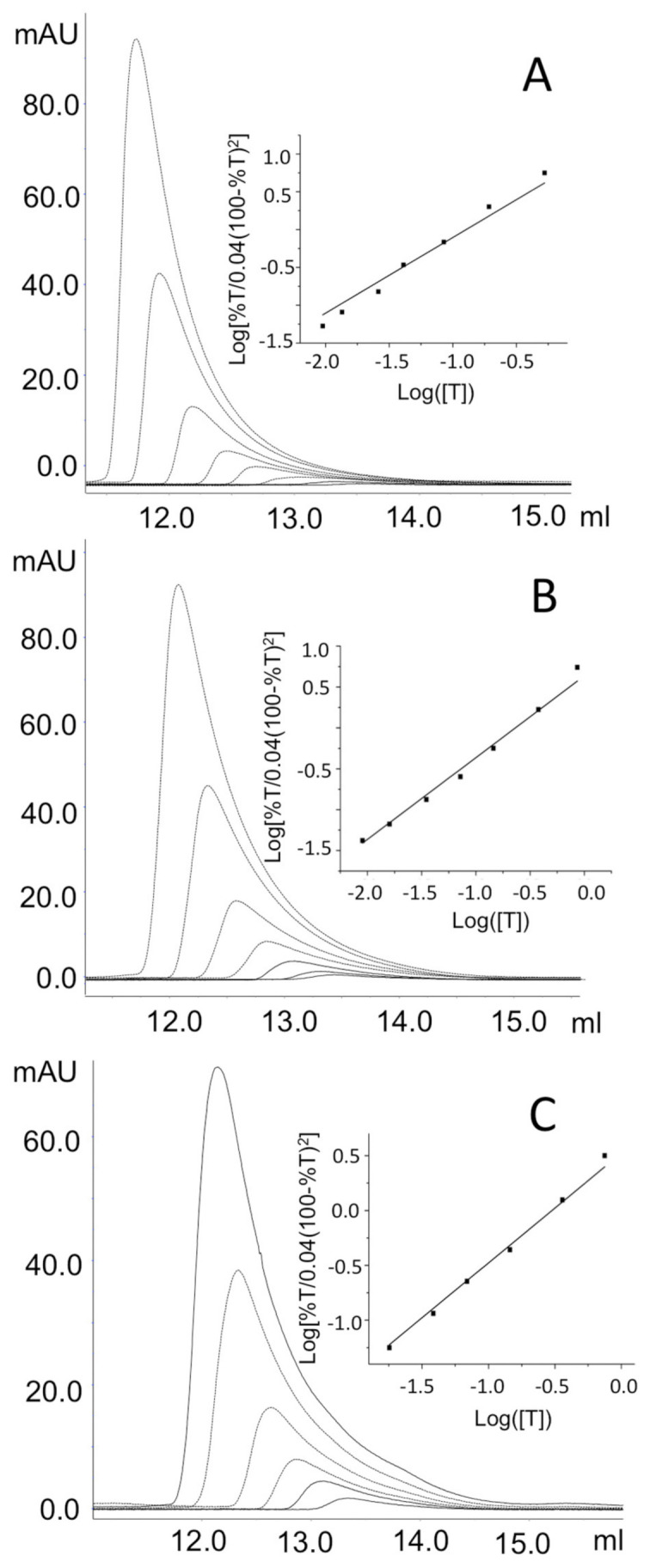
Analysis of the tetramer-dimer equilibrium of hOAT wild type, G51D and G121D variants by SEC. Chromatographic profiles obtained using a Superdex 200 10/30 Increase column equilibrated in HEPES 50 mM pH 8.0, NaCl 150 mM, PLP µM of (**A**) OAT wild-type, (**B**) G51D variant and (**C**) G121D variant from 10 to 0.1 µM protein concentration. Inset panels show the plots of log[%T/0.04(100 − %T)^2^] vs. log([T]), when [%T/0.04(100 − %T)^2^] = 1, [T] = K_Dtet-dim_. Chromatograms were analyzed by Unicorn v7.3 software (GE Healthcare, Chicago, IL, USA); The linear plots were obtained using Origin9 software (OriginLab, Northampton, MA, USA).

On the basis of these results, the K_Dtet-dim_ can be considered >10 µM for these variants (Table 1). The K_Dtet-dim_ values of the remaining two variants, G51D and G121D, and of the hOAT wild-type were evaluated by analyzing the elution profile at different enzyme concentration (Figure 6), as described in the Materials and Methods. Results indicate that the G51D and G121D mutations affect the tetramer–dimer equilibrium, even if to a minor extent with respect to the other analyzed variants. In fact, the K_Dtet-dim_ of G51D and G121D resulted 0.24 ± 0.01 µM and 0.30 ± 0.01 µM, respectively, values that are about twice that of wild-type hOAT (Table 1).

Although none of the substitutions affected residues directly involved in the dimer–dimer interaction, our data indicate that, even if to a different degree, all mutations affect the K_Dtet-dim_ values. This could be explained considering that, as mentioned above, the dimer–dimer surface includes a region along the junction line of the monomers (Figure 1B). Based on this observation, the structural alteration of the monomer–monomer interface could indirectly affect the dimer–dimer interaction site. In support of this hypothesis, the variants that exhibit the higher values of ΔΔG, i.e., R154L, P199Q, and T181M, show consistent alterations of the K_Dtet-dim_ values with respect to that of the wild-type enzyme (Table 1). In addition to the effect on the ΔΔG of interaction of the monomers, the relative position of the amino acid substitutions with respect to the dimer–dimer interface should be considered to explain the impact on the K_Dtet-dim_ value. Arg154, Tyr158, and Pro199 are located closer to the dimer–dimer interface, and Thr181, although lying close to this region, is in a more inner position, while Gly51 and Gly121 are far away from the dimer–dimer interface (Figure 2A). Thus, it is not surprising that R154L, Y158S, and P199Q mutations have a stronger effect on the hOAT tetramerization. Moreover, the high predicted ΔΔG value of monomer–monomer binding together with the consistent structural alterations of the T181M variant revealed by the spectroscopic analyses could account for a perturbation of the tetramerization site due to remote effects transmitted through the monomer–monomer interface. In contrast, the G51D and G121D variants exhibit a low value of ΔΔG of dimerization and only a slight increase of the K_Dtet-dim_ value. It should be noted that although these speculations are relevant for the identification of the structural elements critical for the tetramerization of the hOAT, they do not seem having any impact on OAT catalysis, considering that the catalytic unit of hOAT is a dimer [5]. Nevertheless, it cannot be excluded that the alteration of the tetramer↔dimer equilibrium contributes to the GA pathogenesis.

### 2.5. Impact of the G52D, G121D, R154L, Y158S, T181M and P199Q Mutations on the Kinetic Features of hOAT

To understand if the mutations taken in consideration in this study affect the transaminase activity, the steady-state kinetic parameters of the T181M, P199Q, Y158S variants were measured and compared with those of wild-type hOAT (Table 2). The variants, bearing mutations mapping to the large domain of the enzyme, exhibit a decrease in the *k*_cat_ and k_cat_/K_m_ values, being those of the P199Q the most pronounced ones. The reduction of catalytic efficiency is due to (i) the high K_m_ values for L-Orn (~14-fold) and for α-KG (~4-fold) of the T181M variant, (ii) the high values for α-KG (~22-fold) and L-Orn (~3-fold) of the P199Q variant, and (iii) the high K_m_ value for α-KG (~8-fold) of the Y158S variant. It should be noted that the latter variant displays a substrate inhibition kinetics in the presence of increasing L-Orn concentrations giving a K_i_ equal to 76 ± 13 mM (Figure S3). A substrate inhibition was already observed for the C394Y variant [10]. The functional defects of these pathogenic variants are consistent with their structural defects, shown by the decrease of their visible and near-UV CD signals (Figure 4B) as well as by their values of optical activity. In particular, the P199Q and Y158S mutations could interfere with the local conformation of interfacial loops, while the T181M mutation could generate a hindrance between the two subunits. Again, it should be noted that Thr181 is adjacent to Arg180, a residue critical for catalytic activity [9]. On the other hand, the finding of a lack of detectable transaminase activity of the R154L variant at 2 µM concentration in the presence of 100 μM PLP concentration indicates that this variant is catalytically incompetent at least under the standard assay conditions. Absorption spectra registered immediately after addition of 100 mM L-Orn to 5 μM wild-type show the complete conversion of the 420 nm absorbance band to one centered at 330 nm [8]. In contrast, addition of 100 mM L-Orn to 5 μM R154L leads within 20 min to an incomplete conversion of the 420 nm absorbance band to one at 325 nm band (Figure S4A). It is hard to define the structural alterations responsible for the catalytic inability of the R154L variants. From the in silico analysis, the mutation is predicted to mainly affect the loop 193–203, an important junction region between the monomers, which contributes to protect the cleft between the two active sites from the solvent. It can be speculated that changes of the microenvironment between the catalytic sites could account for the internal aldimine alteration and the impairment of the catalysis. A peculiar kinetic behavior is displayed by the G51D and G121D variants, which share the following similar features: (1) similar values of optical activity, close to that of the wild-type enzyme, (2) an expression level in the bacterial lysate similar each other and lower than that of the wild-type hOAT, and (3) similar values of T_m_, K_Dtet-dim_ and ΔΔG values. Figure S5 shows the typical assay for production of the dihydroquinazolium derivative of P5C. The rate of product formation decreased exponentially and after 300 sec the activity was near zero, with only 40–50 µM P5C being formed. The apparent rate of activity loss was around 0.7 ± 0.09 min^−1^ and 1.3 ± 0.2 min^−1^ for the G121D and G51D, respectively. Since a large excess of L-Orn and α-KG were present in the assay reaction mixture, the termination of activity should not be the result of exhaustion of substrates. However, it could be the result of product inhibition. To test if this was the reason of the loss of activity, we added a second and equal amount of the variant to the reaction mixture. The activity resumed at a similar rate was observed with the first addition of the enzyme, showing that product inhibition was not the reason for the loss of activity of these variants. Further experiments were conducted in order to identify the reason of the inactivation. Like the wild-type enzyme, absorption spectra immediately registered after addition of 100 mM L-Orn to 5 μM G51D or G121D show the complete conversion of the 420 nm absorbance band to one centered at 325 nm (Figure S4B,C). Furthermore, the wild-type (2 µM) or the G51D or G121D enzymes (2 µM) was incubated with 100 mM L-Orn for 2 min or 10 min, respectively. The reaction mixtures were then denatured with TCA at 10% final concentration and, after removal of the precipitated protein by centrifugation, the supernatants were subjected to HPLC analysis as described under “Materials and Methods”. This analysis reveals that ~97% of the original PLP content of the wild-type and ~95% of the G51D and G121D variants were transformed into PMP. Overall, these data indicate that, like for the wild-type, the half-reaction of the variants generates PMP, and that the G51D and G121D mutations do not hamper the conversion of the enzyme from PLP to the PMP form. Again, after incubation at 25 °C of the wild-type (2 µM) for 2 min or the G51D or G121D enzymes (2 µM) with 100 mM L-Orn and 100 mM α-KG for 10 min, the reaction mixtures were (i) denatured and centrifuged to remove the protein or (ii) transferred to an Amicon Ultra device (cutoff 10 kDa) and the filtrates treated with TCA at 10 % final concentration. HPLC analyses of the solutions obtained by these treatments were performed. For treatment (i) we found, with respect to the original PLP content, peaks corresponding to 70% and 30% of PLP and PMP for the wild-type, respectively, and peaks corresponding to ~8% and ~92% of PLP and PMP, for the G51D and the G121D variants, respectively. For treatment (ii) the filtrate of the wild-type contains undetectable PLP and~6% of PMP, while that of the G51D and G121D variants contain less than 2% of PLP and ~98% of PMP. Altogether, these results strongly suggest that their inactivation could be due to the release of PMP during the overall transaminase reaction, thus indicating that in the G51D and G121D variants PMP binding is greatly affected. On the basis of the in silico inspection, G51D and G121D mutations could affect the active site flexible regions 107–119 and 319–327. The latter includes the Thr322, whose side chain engages the phosphate group of the coenzyme of the neighboring subunit by an H-bond linkage [4]. The impairment of such interaction could explain the release of the PMP which, unlike the PLP, is held only by non-covalent interactions. These data also suggest that a conformational change occurs during the conversion of hOAT from PLP to PMP form. 

## 3. Materials and Methods

### 3.1. Materials

L-Orn, α-KG, 2-aminobenzaldehyde, dimethyl sulphoxide, isopropyl-β-D-thiogalactoside, PLP, phenylmethylsulfonyl fluoride (PMSF) were purchased from Merck & Co (Rahway, NJ, USA), while 1,8-anilino-naphthalene sulfonic acid (ANS) was purchased from Molecular Probes (Eugene, OR, USA). All other chemicals were of the highest purity available.

### 3.2. Computational Studies

The coordinate files of the dimer and the tetramer of hOAT were previously generated starting from the solved structure the enzyme free of ligand, available on the Protein Data Bank (pdb file 1OAT) [5]. The surface of the monomer–monomer interface and of the dimer–dimer interface of the dimeric and tetrameric assembly, respectively, were calculated by PDBePISA online tool (https://www.ebi.ac.uk/msd-srv/prot_int/cgi-bin/piserver, accessed on 20 October 2014). The inspection of the monomer–monomer and the dimer–dimer interface contacts were performed by Maestro software v13.3 (Schrödinger, New York, NY, USA). The ΔΔG of dimerization of each pathogenic variant was calculated through the BindProfX server (https://zhanggroup.org/BindProfX/ [18], accessed on 22 December 2020), using the hOAT dimer coordinate file as template. BindProfX was chosen because it is specific for the analysis of protein-protein interface alterations. Moreover, the algorithm combines conservation scores from pairs of protein-protein interaction surfaces sequence profiles with the FoldX physics-based potential to predict changes in binding affinity upon mutations in the form of ΔΔG (change in free energy of binding) values. The hOAT dimer coordinate file was used to determine the amino acids conservation score, which was calculated by the Consurf Server (https://consurf.tau.ac.il/, accessed on 26 January 2023), using the PSI-BLAST as the homolog search algorithm between 35% and 95% of homology, the UniProt database, and CLUSTALW alignment software. Non-redundant homologous sequences were identified and used to generate the amino acid conservation score, expressed on a scale of 1 (not conserved) to 9 (highly conserved). The in silico mutagenesis was carried out by Maestro software mutation tool. An energy minimization process in explicit solvent was carried out on the hOAT structure before and after the mutagenesis using the GROMACS v4.6.3 software. The variants model structures underwent two minimization steps (with and without restraints), alternating Steepest Descent and Conjugate Gradient algorithms.

### 3.3. Site Directed Mutagenesis

The pOAT expression vector, previously obtained by cloning the 26–439 coding sequence of hOAT in the pET43a expression vector [5], was used as template to generate the expression vectors of the G51D, G121D, Y158S, T181M and P199Q pathogenic variants. The mutagenesis reactions were performed by means of the *Quick-Change II mutagenesis kit* (Agilent Technologies, Santa Clara, CA, USA) using the mutagenetic primers reported in (Supplementary Table S1) and its complements. The mutations were confirmed by DNA sequence analysis of the whole coding region. The expression vector of the R154L variant was previously obtained [10].

### 3.4. Expression and Purification of OAT Variants

hOAT recombinant variants were purified from *E. coli* expression and subsequent cell lysis following the steps previously described [5]. The soluble fraction of the lysate was loaded on a DEAE Sepharose 26/20 equilibrated with 20 mM sodium phosphate buffer, pH 7.6. Then, a gradient from 20 to 200 mM sodium phosphate buffer, pH 7.6, was applied. Under these conditions, both hOAT wild-type and pathogenic variants eluted at a concentration of sodium phosphate between 110 and 160 mM. By using an Amicon Ultra 15 unit (Merck & Co, Rahway, NJ, USA), fractions containing the hOAT enzymes were concentrated and then loaded on a Superdex 200XK 16/60 column (GE Healthcare, Chicago, IL, USA) equilibrated in 50 mM HEPES pH 8.0, 200 mM NaCl. Purified proteins were finally concentrated and stored at −20 °C. The purity of each preparation assessed by SDS-PAGE was >95%. 

### 3.5. Western-Blot

Soluble fractions of the *E. coli* cell lysate expressing the wild-type and variants OAT were loaded on a 12% SDS-PAGE gel (15 µg each), then they were transferred on a PVDF membrane and immunoblotted with anti hOAT antibody 1:1000 (OriGene Technologies Inc., Rockville, MD, USA) and horseradish peroxidase-conjugated anti-IgG antibody (1:5000). A chemiluminescence kit (Thermo Fisher Scientific Inc., Waltham, MA, USA) was adopted to visualize and enhance immunocomplexes. ImageJ software (National Institute of Health, Bethesda, MD, USA) was used to quantify the relative bands intensity. 

### 3.6. Enzyme Activity Assays

hOAT activity was measured by spectroscopic quantification of the dihydroquinazolium derivative of P5C after incubation with 2-aminobenzaldehyde. L-Orn and α-KG were paired to determine the kinetic parameters for the overall transamination. Purified protein was incubated in 50 mM HEPES pH 8.0, 150 mM NaCl, in the presence of 50 µM PLP at 25 °C at different substrate concentration at a fixed saturating co-substrate concentration. Data obtained were then fitted to the Michaelis-Menten equation. Substrate inhibition kinetics of the Y158S variant was instead analyzed according to the following equation:

$$\frac{v}{E_{t}}=\frac{k_{\mathrm{cat}}}{\left(1+\left(\frac{K_{M}}{S}\right)+\left(\frac{S}{K_{i}}\right)\right)}$$

where E_t_ represents the total enzyme concentration, S the substrate concentration, and K_i_ the inhibition constant.

### 3.7. Spectroscopic Measurements and Thermal Stability 

All spectra, with the exception of the far-UV CD spectra carried out in 5 mM HEPES pH 8.0, 15 mM NaCl, were performed in 50 mM HEPES pH 8.0, 150 mM NaCl at 25 °C. A Jasco V-550 spectrophotometer with 1 cm length quartz cuvettes was adopted to register the absorption spectra at 6 µM enzyme concentration in the absence or in the presence of 100 mM L-Orn. Near-UV and visible CD spectra were registered at 6 µM protein concentration in the presence of 20 µM exogenous PLP by a Jasco J-710 spectropolarimeter equipped with a thermostatically controlled compartment, using 1 cm path length quartz cuvettes. Each measurement relied upon three spectra recorded at a scan speed of 50 nm/min with a bandwidth of 2 nm and averaged automatically. Far-UV CD spectra were recorded at a protein concentration of 1 µM and a pathlength quartz cuvette of 0.1 cm, Fluorescence measurements were carried out at a protein concentration of 1 µM in the presence of 10 µM PLP. Intrinsic fluorescence emission spectra were taken upon excitation at 280 nm, while ANS emission spectra were taken upon excitation at 365 nm of samples at 1 µM enzyme concentration after incubation for 1 h on ice in presence of 20 µM ANS. Thermal denaturation profiles were registered at 1 μM enzyme concentration in the presence of 20 µM PLP by monitoring the far-UV CD signal at 222 nm over a temperature gradient from 25 to 90 °C with a slope of 1.5 °C/min. All the spectra and the unfolding curves were analyzed by Spectra Manager v1.43 software (Jasco Europe, Cremella, Italy).

### 3.8. Analytical Size-Exclusion Chromatography (SEC)

For SEC analyses, an AktaPure FPLC system (GE Healthcare, Chicago, IL, USA) and a Superdex 200 Increase 10/300 GL column were used. The column was equilibrated and run in 50 mM HEPES, 150 mM NaCl, pH 8.0 containing 20 µM PLP. hOAT wild-type and the pathogenic variants were diluted at different enzyme concentration from 0.5 to 10 µM in the running buffer and incubated at 25 °C for 20 min. Then, 100 µL were loaded on the column and eluted at a flow rate of 0.3 mL/min. Resulting chromatographic profiles were analysed by the Unicorn v7.3 software (GE Healthcare, Chicago, IL, USA). The K_Dtet-dim_ values of the wild-type OAT, G51D and G121D variants were calculated following the methods of Manning at al [19], as previously applied for hOAT [5].

### 3.9. HPLC Analysis of the Coenzymes

For the analyses of the coenzymes upon reaction with the substrates, the following experiments were performed. The enzyme at 2 µM concentration was incubated with 100 mM L-Orn at 25 °C for 10 min in HEPES 50 mM pH 8.0, NaCl 150 mM. The reaction mixture was then deproteinized by the addition of TCA at 10 % final concentration and centrifuged to remove the protein. When the enzyme at 2 µM concentration was incubated with 100 mM L-Orn and 100 mM α-KG for 10 min at 25 °C, the reaction mixture was filtered through an Amicon Ultra15 concentrator, and TCA 10% was added. PLP and pyridoxamine

e 5′-phoshate (PMP) were quantify by the analysis of samples (200 µL) loaded on a C18 (250 mm × 4.6 mm) column connected to a Jasco PU-2080 Plus HPLC control system (Jasco Europe, Cremella, Italy). The eluent was 50 mM potassium phosphate buffer, pH 2.35, at a flow rate of 1 mL/min. A Jasco UV-2075 Plus detector set at 295 nm was employed. Peaks corresponding to PMP and PLP were integrated using the Jasco ChromNav v2.01.01 software (Jasco Europe, Cremella, LC, Italy). Standard curves of peak area were prepared using commercially available PLP or PMP.

## 4. Conclusions

The paper describes the structural and/or functional effects generated by the pathogenic mutations of the interfacial monomer–monomer residues, Gly51, Gly121, Arg154, Tyr158, Thr181 and Pro199, of hOAT. With respect to the wild-type enzyme, all the G51D, G121D, R154L, Y158S, T181M, and P199Q variants show, even if to a different degree, changes in their tertiary and quaternary structures, in the thermal stability as well as in their catalytic activity. Furthermore, evidence is provided that the R154L, Y158S, T181M, and P199Q variants involving residues mapping to the large domain and near to the junction point of the dimer–dimer interaction display, as compared to the wild-type enzyme, a remarkably decreased T_m_ value, a marked increased K_Dtet-dim_ value as well as a consistent change of their PLP microenvironment. On the other hand, the G51D and G121D variants relative to residues belonging to the N-terminal segment and far away from the dimer–dimer interface exhibit less pronounced alterations of their T_m_, K_Dtet-dim_ values and their PLP-binding site. Considering the different location of these mutated residues in the monomer–monomer surface and their different ΔΔG values of monomer–monomer binding, it is reasonable to suggest that a marked perturbation of the monomer–monomer interaction interface could have an effect not only on the thermal stability and on the coenzyme environment but also on the tetramerization process. The latter effect is in line with the bioinformatics predictions. 

As far as the impact of the studied mutations on catalytic activity is concerned, it was found that (i) the Y158S, T181M, and P199Q variants show a decrease of the *k*_cat_ value (ranging from 5 to 130-fold), (ii) the R154L variant exhibits undetectable transaminase activity at least under the assay conditions, and (iii) the G51D and G121D display a loss of the of linearity in the formation of the product within 1 min of reaction. The catalytic defect(s) of the Y185S, T181M, and P199Q variants seem to be ascribable to the perturbation of their active site. In contrast, it is not easy to trace back the catalytic defect of the G51D, R154L, and G121D to structural defect(s). Our data suggest that the R154L mutation affects the half-transamination reaction, while the G51D and G121D mutations appear to exert an effect on the overall transamination, due to the inability to maintain PMP bound to the enzyme in the presence of L-Orn and α-KG. Further experiments will be necessary to define the structural features at the basis of the loss of a proper and functionally active conformation of hOAT caused by the G51D, G121D and R154L pathogenic mutations. Moreover, it is reasonable to suggest that the Tyr158→Ser and Thr181→Met substitutions, significantly affecting the thermal stability and the expression level of the enzyme, could induce a folding defect. On the basis of this hypothesis, a chaperone role of the coenzyme, already seen for rare diseases involving B6-enzymes [20], could explain the reason of the observed responsiveness to pyridoxine administration for patients bearing these mutations [13,21]. Overall, these biochemical and bioinformatic studies allow not only to identify the relevance of interfacial residues on the structural and/or functional features of hOAT but also to characterize the enzymatic phenotype of GA patients bearing these mutated residues.

## Figures and Tables

**Table 1:** ΔΔG, ΔT_m_ and K_Dtet-dim_ of wild-type hOAT and variants. Predicted values of ΔΔG of dimerization calculated by the BindProfX. ΔT_m_: difference between the T_m_ value of the hOAT wild-type (67.8 °C) and that of the variants; K_Dtet-dim_: tetramer↔dimer equilibrium of dissociation constant of hOAT wild-type and the variants.

Enzymatic Species	ΔΔG (kcal/mol)	ΔT_m_ (°C)	K_Dtet-dim_ (µM)
OAT wild-type	0	0	0.13 ± 0.01
G51D	1.95	−0.5	0.24 ± 0.01
G121D	3.83	−1.3	0.30 ± 0.01
R154L	8.68	−12	>>10
Y158S	2.07	−12	>>10
T181M	11.52	−8.9	>10
P199Q	10.82	−10.5	>>10

**Table 2:** Steady-state kinetic parameters of wild-type and variants OAT enzymes.

Enzyme	Substrate	Cosubstrate	*k_cat_* (s^−1^)	K_M(L-Orn)_ (mM)	K_M(α-KG)_ (mM)	*k_cat_*/K_M_ (mM^−1^s^−1^)
OAT WT [5]	L-Orn	α-KG	34.9 ± 0.6	6.5 ± 0.4		5.4 ± 0.3
	α-KG	L-Orn	35.7 ± 0.7		3.9 ± 0.5	9.1 ± 1.2
T181M	L-Orn	α-KG	6.8 ± 0.3	91 ± 10		0.075 ± 0.009
	α-KG	L-Orn	7.2 ± 0.4		14.7 ± 2.8	0.49 ± 0.10
P199Q	L-Orn	α-KG	0.25 ± 0.01	18.8 ± 1.9		1.3 × 10^−3^ ± 2 × 10^−4^
	α-KG	L-Orn	0.39 ± 0.03		87.6 ± 0.5	4.4 × 10^−4^ ± 3 × 10^−5^
Y158S	L-Orn	α-KG	1.55 ± 0.13	3.4 ± 0.7		0.45 ± 0.10
	α-KG	L-Orn	2.19 ± 0.14		31.0 ± 6.4	0.07 ± 0.02

## Data Availability

Not applicable.

## Supplementary Materials

The following supporting information can be downloaded at: https://www.mdpi.com/article/10.3390/ijms24043369/s1.

## Abbreviations

Human ornithine aminotransferase, hOAT; Pyridoxal 5′-phosphate, PLP; Pyridoxamine 5′-phosphate, PMP; L-Ornithine, L-Orn; α-ketoglutarate, α-KG, Gyrate atrophy of choroid and retina, GA; Tetramer-dimer equilibrium dissociation constant, (K_Dtet-dim_); Δ1-pyrroline-5-carboxylate, P5C; 1,8-anilino-naphtalene sulfonic acid, ANS; Size-exclusion chromatography, SEC.
