# Supplementary material for: Biochemical and Bioinformatic Studies of Mutations of Residues at the Monomer–Monomer Interface of Human Ornithine Aminotransferase Leading to Gyrate Atrophy of Choroid and Retina
Source: Int J Mol Sci. 2023 Feb 8;24(4):3369. doi: 10.3390/ijms24043369 (PMC9967328; doi:10.3390/ijms24043369)
Supplement: Supplementary file 1 [file ijms-24-03369-s001.zip › ijms-2147025-supplementary.pdf]

Table S1. Site directed mutagenesis primers

Mutation	Oligonucleotides
G51D	5’ GAAAGGGAATATAAGTATGAGCCACAACTACATCC 3’
G121D	5’ GCTTTCTATAATAACGTACTTGATGAATATGAGGAGTATATAC 3’
Y158S	5’ CTCGTAAGTGGGGCTCTACCGTGAAGGGCATTC 3’
T181M	5’ GAACTTCTGGGGTAGGATGTTGTCTGCTATCTCC 3’
P199Q	5’ GTTACGATGGTTTTGGACAATTTATGCCGGGATTCGAC 3’

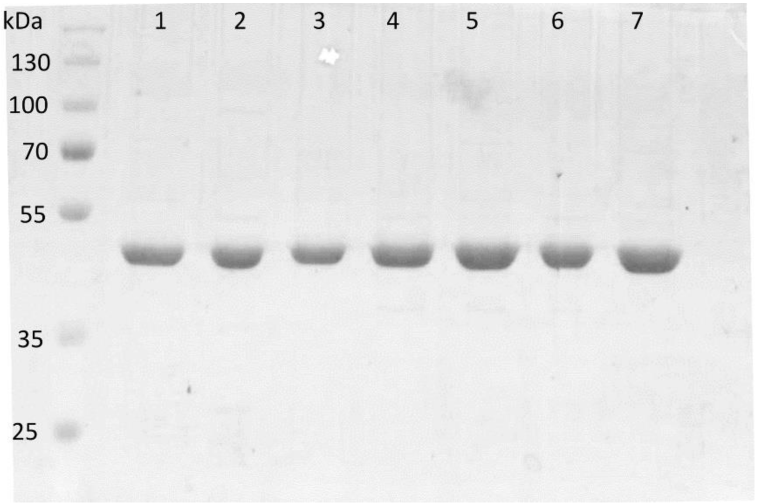

Figure S1. SDSpage analysis of hOAT variants in purified form. After thermal denaturation for 5 min at 95°C in electrophoresis sample buffer, samples containing 4 µg of OAT wild type (lane 1), G51D (lane 2), G121D (lane 3), R154L (lane 4), Y158S (lane 5), T181M (lane 6) and P199Q (lane7) in purified form were loaded on a 12% SDS polyacrylamide gel and stained with Coomassie dye.

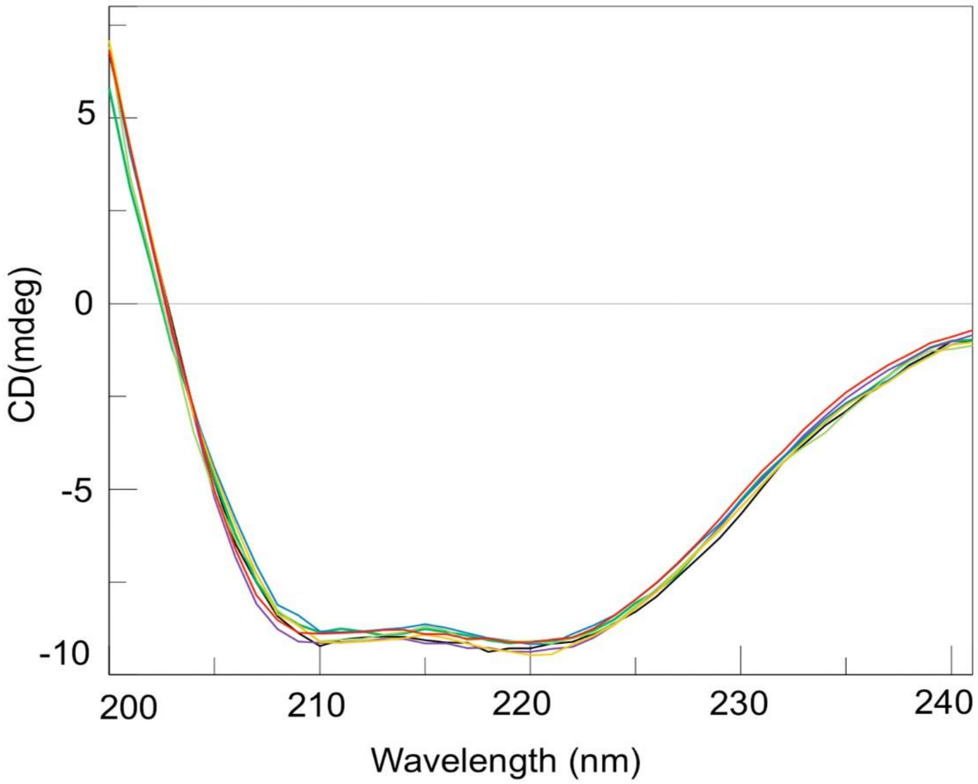

Figure S2. FarUV CD spectra of wild type and variants OAT. Far UV spectra of wild type OAT (black), G51D (dark green), G121D (blue), R154L (red), Y158S (ligh green), T181M (purple) and P199Q (orange). All the spectra were registered at 1 µM enzyme concentration in 5 mM HEPES pH 8.0, 15 mM NaCl in the presence of 10 µM exogenous PLP. Spectra were analyzed by Spectra Manager software (Jasco).

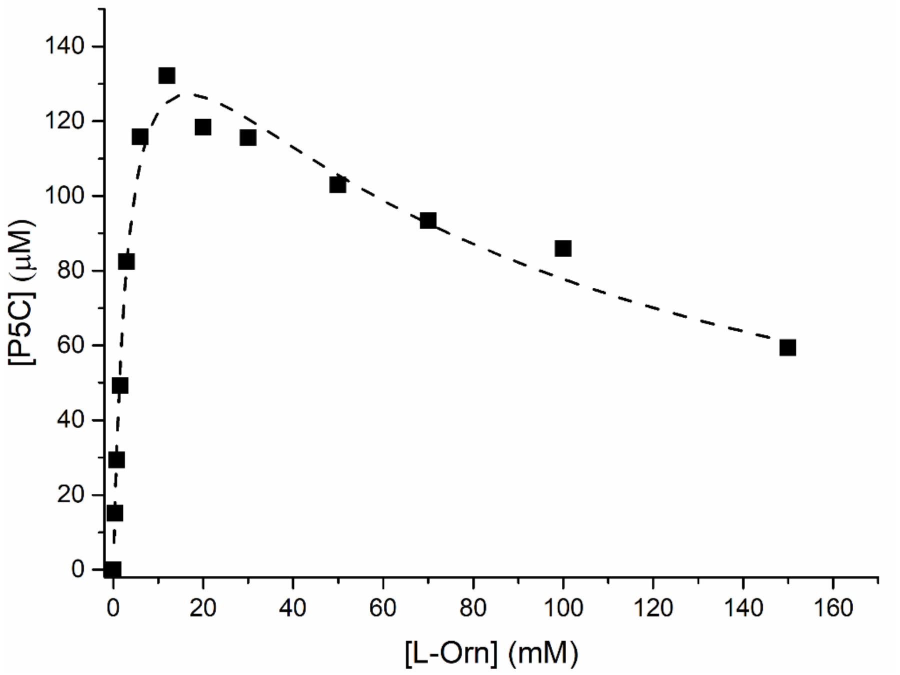

Figure S3. Substrate inhibition kinetic of Y158S variant. Kinetic of substrate inhibition by L-Orn of Y158S in the presence of 100 mM αKG and PLP 50µM in HEPES 50 mM pH 8.0, NaCl 150 mM at 25°C. Fitting was performed using Origin9 software (OriginLab).

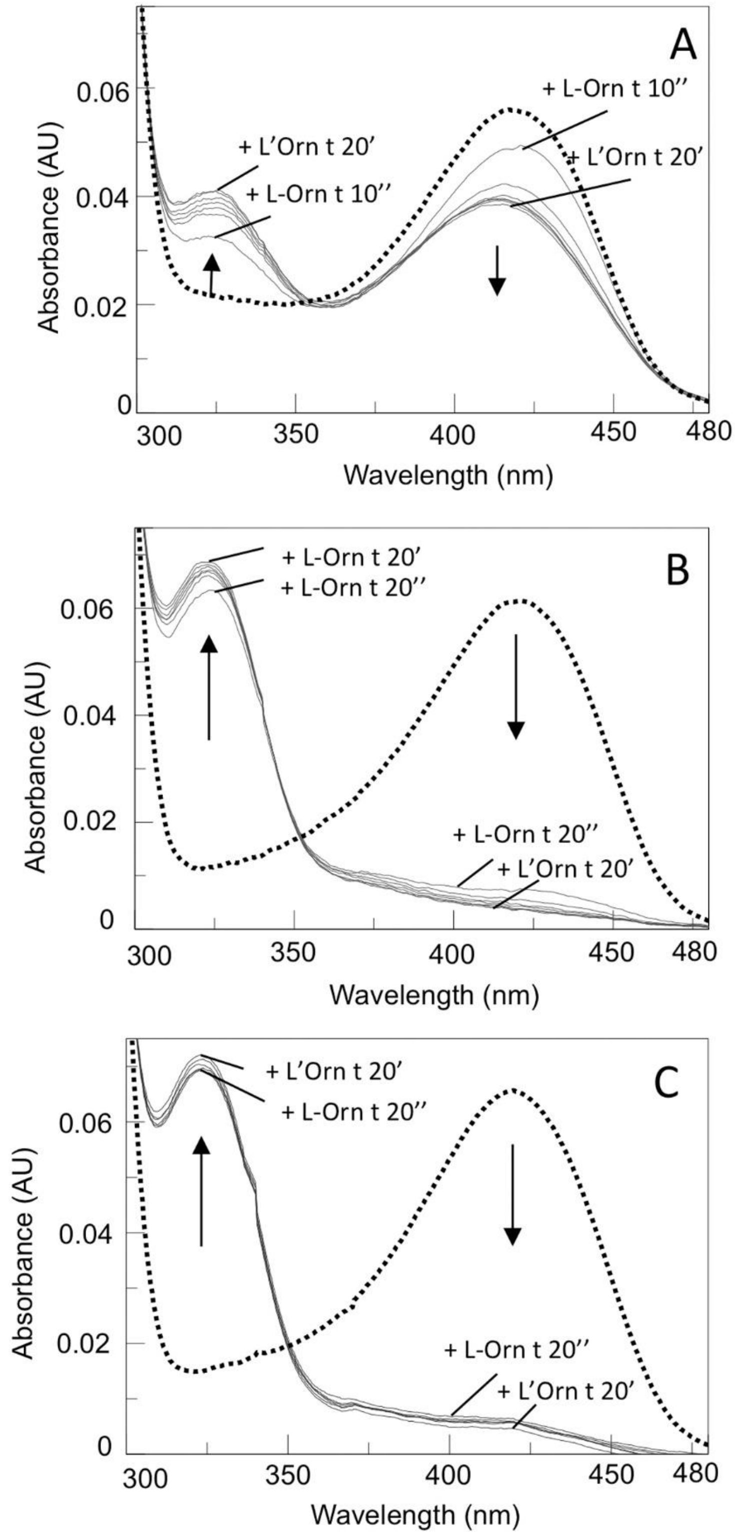

Figure S4. Absorbance spectra in the presence of L-Orn. UV-vis absorbance spectra of 5 µM (**A**) R154L, (**B**) G51D and (**C**) G121D, before (----) and after (—) the addition of 100 mM L-Orn in HEPES 50 mM buffer pH 8.0, NaCl 150 mM.

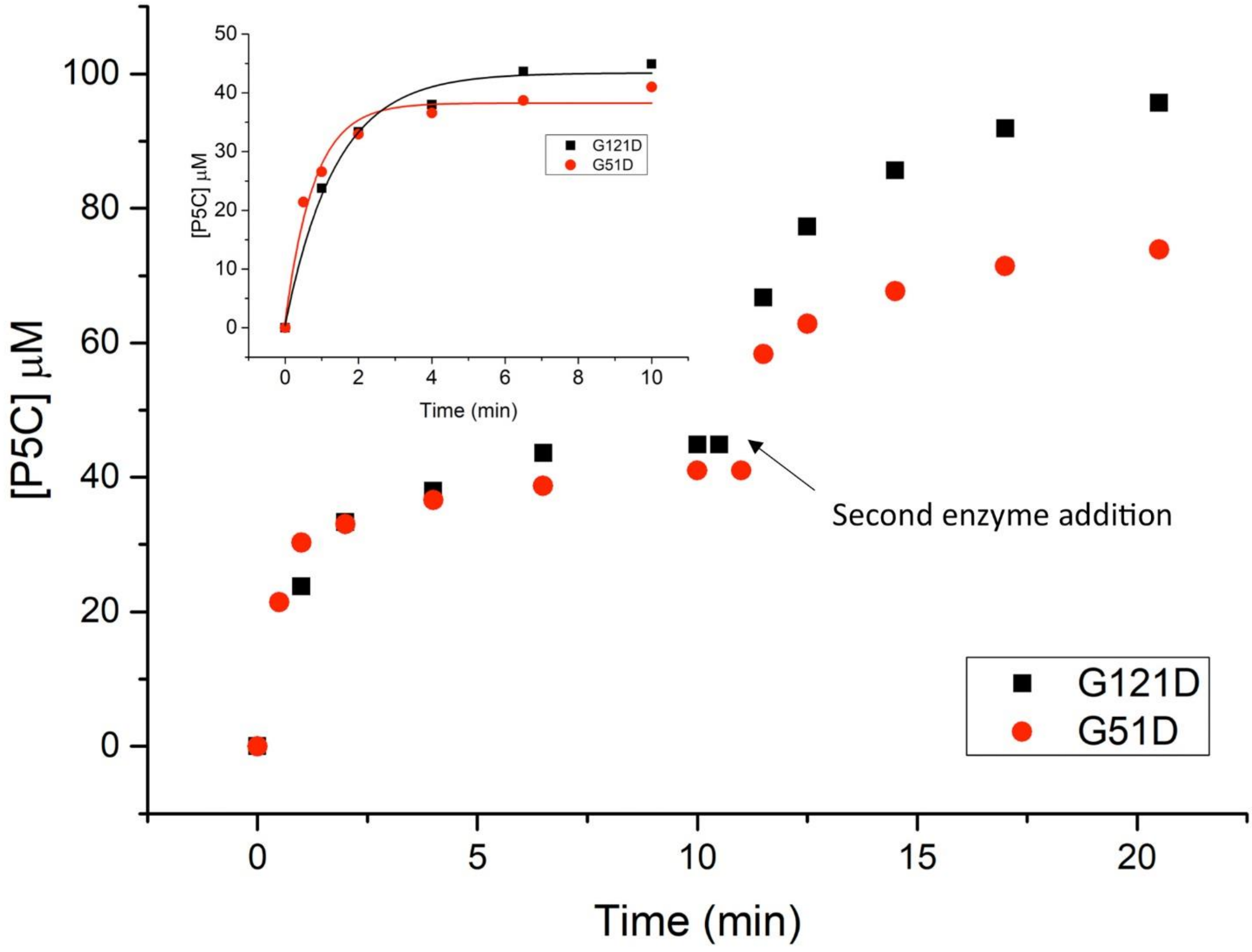

Figure S5. Inactivation kinetic of G51D and G121D variants. Kinetic of P5C formation during the incubation of G51D or G121D with 100 mM L-Orn and 100 mM αKG at 25°C in HEPES 50mM pH 8.0, NaCl 150 mM. The product formation was followed by measuring the absorbance at 440 nm of the dihydroquinazolium derivative of P5C; the second enzyme addition is indicated. **Inset:** inactivation exponential curves of G51D and G121D; the fitting to a first order exponential equation was performed by Origin9 software (OriginLab).
